# High Genetic Stability of Dengue Virus Propagated in MRC-5 Cells as Compared to the Virus Propagated in Vero Cells

**DOI:** 10.1371/journal.pone.0001810

**Published:** 2008-03-19

**Authors:** Chia-Chyi Liu, Shiang-Chi Lee, Michael Butler, Suh-Chin Wu

**Affiliations:** 1 Institute of Biotechnology, Department of Life Science, National Tsing Hua University, Hsinchu, Taiwan; 2 Department of Microbiology, University of Manitoba, Winnipeg, Manitoba, Canada; Federal University of Sao Paulo, Brazil

## Abstract

This work investigated the replication kinetics of the four dengue virus serotypes (DEN-1 to DEN-4), including dengue virus type 4 (DEN-4) recovered from an infectious cDNA clone, in Vero cells and in MRC-5 cells grown on Cytodex 1 microcarriers. DEN-1 strain Hawaii, DEN-2 strain NGC, DEN-3 strain H-87, and DEN-4 strain H-241 , and DEN-4 strain 814669 derived from cloned DNA, were used to infect Vero cells and MRC-5 cells grown in serum-free or serum-containing microcarrier cultures. Serum-free and serum-containing cultures were found to yield comparable titers of these viruses. The cloned DNA-derived DEN-4 started genetically more homogeneous was used to investigate the genetic stability of the virus propagated in Vero cells and MRC-5 cells. Sequence analysis revealed that the DEN-4 propagated in MRC-5 cells maintained a high genetic stability, compared to the virus propagated in Vero cells. Amino acid substitutions of Gly_104_Cys and Phe_108_Ile were detected at 70%, 60%, respectively, in the envelope (E) protein of DEN-4 propagated in Vero cells, whereas a single mutation of Glu_345_Lys was detected at 50% in E of the virus propagated in MRC-5 cells. Sequencing of multiple clones of three separate DNA fragments spanning 40% of the genome also indicated that DEN-4 propagated in Vero cells contained a higher number of mutations than the virus growing in MRC-5 cells. Although Vero cells yielded a peak virus titer approximately 1 to 17 folds higher than MRC-5 cells, cloned DEN-4 from MRC-5 cells maintained a greater stability than the virus from Vero cells. Serum-free microcarrier cultures of MRC-5 cells offer a potentially valuable system for the large-scale production of live-attenuated DEN vaccines.

## Introduction

The four dengue serotype viruses (DEN-1 to DEN-4) (genus *flavivirus*, family *Flaviviridae*) are single stranded, positive-sense RNA viruses that are transmitted to humans primarily by *Aedes aegypti* mosquitoes. The RNA genome contain sequences coding for three structural protein genes (core C, precursor membrane prM, and envelope E), seven non-structural protein genes (NS1, NS2A, NS2B, NS3, NS4A, NS4B, NS5), and the flanking non-translating regions. DEN infection in humans causes a spectrum of illnesses, ranging from dengue fever (DF) to dengue hemorrhagic fever (DHF) and dengue shock syndrome (DSS) [1]. Approximately 50–100 million infections, including 250,000 cases of DHF with fatality rates varying from <1% to 5%, occur every year mostly in tropical and subtropical areas [2]. Dengue is endemic in South-east Asia, where the severe form of DHF/DSS frequently associated with secondary DEN infections is the major cause of hospitalization of young children [3]. A safe and effective dengue vaccine is still not available for prevention of DEN infection.

The current DEN immunization strategy favors the use of live-attenuated vaccines which offer the advantage of a single dose, delivering a complete set of protective antigens and achieving a long-lasting immunity [4]. Further, the use of a tetravalent DEN vaccine against each of the four serotypes would have the possibility to minimize the risk of severe dengue [4]. Wild type DEN-1, DEN-2, DEN-3, DEN-4 strains have been attenuated by serial passage in primary dog kidney cells (or monkey kidney cells) [5]–[7] and final vaccines were produced in fetal rhesus lung cells or Vero cells [8]–[10]. Extensive clinical trials have demonstrated that each monovalent DEN vaccine appeared to be immunogenic and safe [6]–[10]. However, data obtained from clinical trials with a tetravalent vaccine formulation have not shown the predicted response, as immune imbalance or reactogenicity occurred for certain DEN serotypes [6], [7]. Several other vaccine researchers also have applied the cDNA cloning added by chimeric virus technology and strategic modifications to generate viruses with the growth restriction phenotype in the genetic background of attenuated virus (for example, using DEN-4 with a deletion in 3′ NTR, attenuated 17D yellow fever vaccine or DEN-2 strain PDK-53) [11]–[20]. All of these cDNA-derived candidate DEN vaccines have been produced in Vero cells, recently certified for vaccine production. Although clinical trails with a few of these monotypic chimeric vaccines appeared to show desirable immunogenicity and reactogenicity, data from studies with such vaccines of other serotypes or a tetravalent vaccine formulation are still not available. Potential replication interference among the four serotype vaccine components that results in immune imbalance remains a concern.

Replication interference could result from loss or increase of infectivity due to genetic instability and accumulation of mutations in certain vaccine components. Passages of DEN viruses or their derived chimeras in Vero cells [21], [22] have been shown to generate mutations specific for host cell adaptation, virus attenuation, or other properties yet to be characterized. Recently, spot-check sequencing in the manufacturing of chimeric DEN-2 PDK-53 vaccine components has detected loss of attenuating mutation markers in a number of seed stocks during first several passages in Vero cells and these vaccine seeds were not appropriate for use [23]. It is also possible that passage of virus in certain cells produces host-cell specific mutations which could contribute to host cells' innate immunity response in the vaccinees. The use of appropriate cell substrates and methods for culturing these cells for production of vaccines represents a critical factor affecting the maximum viral yield, genetic stability, immunogenicity, and ultimately vaccine safety and economic feasibility.

Recently, a serum-free medium has been developed to support the growth of Vero cells on solid microcarriers and produce a high final yield for reovirus [24], Japanese encephalitis virus [25], and enterovirus type 71 [26], [27]. Although an attempt has not been made for producing DEN vaccines, human diploid MRC-5 cells have been already used for the production of several live-virus vaccines such as oral polio, rubella, small pox, and varicella zoster [28]. This study investigated Vero cells and MRC-5 cells for the production of the four wild type DEN serotypes, including DEN-4 derived from an infectious DNA clone. We focused on the processes of cell culture using microcarrier systems which can be adapted for large-scale production and serum-free medium to avoid potential contamination from bovine serum. Currently, several panels of tetravalent DEN vaccine candidates have been derived from viruses of diverse genetic background responsible for growth restriction [13], [16]. In order to simplify interpretation of results, cloned DNA derived wild type DEN-4 was used for comparison of progeny genetic stabilities from passages of the virus in Vero cells and in MRC-5 cells. This strategy using cloned DNA-derived wild type rather than attenuated DEN-4 has been previously used to investigate the genetic mutations of chimeric DEN-4 after Vero cell adaptation [21]. Our present studies show that Vero cells yielded a peak virus titer approximately 1 to 17 folds higher than MRC-5 cells, and DEN-4 propagated in MRC-5 cells maintained a greater stability than the virus propagated in Vero cells. Serum-free microcarrier cultures of MRC-5 cells offer a potentially valuable system for the large-scale production of live-attenuated DEN vaccine.

## Results

### Microcarrier cultures of Vero cells and MRC-5 cells grown in serum-free and serum-containing media

**Figure 1:**
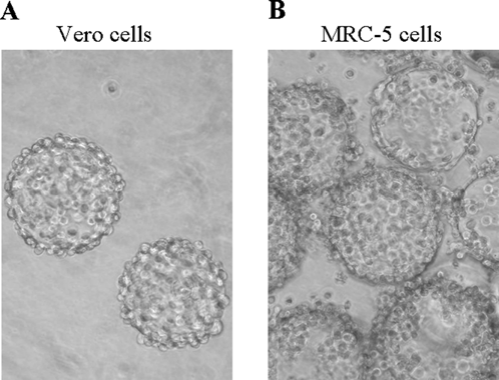
Vero and MRC-5 cell growth on Cytodex 1 microcarriers in serum-free cultures. Microscopic observation shows (A) Vero and (B) MRC-5 cells on microcarriers in M-VSFM after 3 days culture.

Solid microcarriers (Cytodex 1) have been shown to support the growth of Vero cells in either serum-containing or serum-free medium [24]–[27]. To investigate whether microcarriers also support the growth of MRC-5 cells, MRC-5 cells were seeded at an initial density of 3×10^5^ cell/ml in serum-containing medium or 6×10^5^ cells/ml in serum-free medium on microcarriers. Vero cells were also seeded at same density in the respective medium as a control. Three days after incubation, both the Vero cells and MRC-5 cells adhered to the entire surface of the microcarrier beads, reaching confluency as seen microscopically (Fig. 1). The cell density was determined at 1.6×10^6^ cells/ml in serum-free medium or 1.4×10^6^ cells/ml in serum-containing medium for MRC-5 cells, compared to 1.7×10^6^ cells/ml in serum-containing medium or 1.8×10^6^ cells/ml in serum-free medium for Vero cells. Doubling the cell density in serum-free culture was needed to support the cell growth on microcarriers. Increased cell seed lot consumption in serum-free cultures is a continuing problem in vaccine manufacturing processes, particularly in large-scale microcarrier cultures. We are currently optimizing additional components in the M-VSFM medium to reduce the cell density for initial inoculation in serum-free microcarrier cultures (data not shown).

### Production of DEN-1, DEN-2, DEN-3 and DEN-4 in infected Vero cells and MRC-5 cells

Production of DEN-1 strain Hawaii, DEN-2 strain NGC, DEN-3 strain H-87 and DEN-4 strain H-241 in Vero cells and in MRC-5 cells grown in serum-containing and serum-free media was studied. Figs. S2, S3, S4 and S5 show the cell growth and virus replication curves at a multiplicity of infection (MOI) of 0.01 in serum-free and serum-containing media. In each case the cell density decreased gradually during the 10-day period following virus infection, and no appreciable differences were observed between cells growing in serum-free medium and in serum-containing cultures. The virus yields from Vero cells was 1 to 17 folds higher than that from MRC-5 cells for each serotype virus (Table 1), whether in serum-containing or serum-free medium. Compared to DEN-2 NGC, DEN-3 H87 and DEN-4 H241 produced a 10^3^- to 10^4^-fold lower viral yield from Vero cells or a 10^2^- to 10^3^-fold lower yield from MRC-5 cells in serum-containing medium. The serotype difference was probably not responsible for the low viral yields, as the DEN-4 strain derived from cDNA replicated as efficiently as DEN-2. A plausible reason for the unexpected low yields is that these viruses have acquired mutations during passages in cultured cells or in animals. This probability was supported from the analysis of viral yields in similar infections with the wild type DEN-4 derived from a cDNA clone (see next section). Importantly, higher viral yields from Vero cells or MRC-5 cells were consistently observed at 0.3- to 2.6-fold higher for the four DEN serotypes in serum-free medium than in serum-containing media.

**Table 1:** The maximum virus titers for four-serotype DEN virus strains and the cloned DNA-derived DEN-4 viruses in Vero cells and MRC-5 cells on microcarriers, in serum-containing and serum-free media.

	Virus titer (pfu/ml) in Vero cells				
	DEN-1	DEN-2	DEN-3	DEN-4	DEN-4
	(HAWAII)	(NGC)	(H-87)	(H-241)	(DNA clone)
**DMEM+10%FBS**	(1.7±0.1)×10^6^	(1.2±0.0)×10^7^	(1.3±0.1)×10^4^	(2.0±0.2)×10^3^	(1.6±0.3)×10^7^
**M-VSFM**	(4.0±0.1)×10^6^	(2.2±0.0)×10^7^	(3.0±0.1)×10^4^	(2.9±0.2)×10^3^	(1.4±0.1)×10^7^

	Virus titer (pfu/ml) in MRC-5 cells				
	DEN-1	DEN-2	DEN-3	DEN-4	DEN-4
	(HAWAII)	(NGC)	(H-87)	(H-241)	(DNA clone)
**DMEM+10%FBS**	(6.0±0.1)×10^5^	(6.8±0.3)×10^5^	(4.5±0.7)×10^3^	(2.5±0.7)×10^2^	(1.1±0.8)×10^6^
**M-VSFM**	(7.7±0.1)×10^5^	(2.0±0.1)×10^6^	(1.3±0.3)×10^4^	(9.0±2.8)×10^2^	(1.2±0.1)×10^6^

### Propagation of cloned DNA-derived DEN-4 in Vero cells and MRC-5 cells and sequence analysis of progeny DEN-4

DEN-4 strain 814669 recovered from cloned DNA was propagated in microcarrier cultures of both cell lines. Infection with the virus was carried out at the same multiplicity as described earlier. The density of Vero cells or MRC-5 cells decreased steadily during the subsequent 10-day period, indicating that both cells were readily infected (Fig. S5). The viral yields of cloned DNA derived DEN-4 in microcarrier cultures of both cells were comparable to that observed for DEN-2 NGC. There were appreciable differences in the viral yields between cell cultures in serum-containing or serum-free medium (Table 1). Vero cells produced a virus titer of cloned DEN-4 increased by 11 folds (serum-free medium) and 14 folds (serum-containing medium) as compared to MRC-5 cells.

DEN-4 derived from cloned DNA was propagated in the microcarrier cultures of Vero cells or MRC-5 cells in serum-free or serum-containing medium for 4 days. The nucleotide sequence of progeny DEN-4 was determined from a single cDNA clone representing each of the four overlapping regions, designated as W01/W02R, W03/W04R, W05/W06R, W07/W08R, spanning the entire DEN genome. Each progeny DEN-4 sequence was compared with the parent DEN-4 DNA sequence (GenBank accession number: AF375822). The results in Table 2 revealed that there were 18 nucleotide mutations resulting in 13 amino acid changes in DEN-4 propagated in Vero cells using serum-containing medium, and 11 nucleotide mutations resulting in 6 amino acid changes in DEN-4 propagated in Vero cells grown in serum-free medium. In contrast, there was only one nucleotide mutation resulting in one amino acid change in each DEN-4 propagated in MRC-5 cells grown in serum-containing medium or in serum-free medium. None of the amino acid changes was identical between the DEN-4 propagated in Vero cells grown in serum–containing medium or in serum-free medium. Further, these amino acid changes in DEN-4 occurred in nearly all gene segments and the number of mutations, ie., a total of 6 each in NS3 and in NS4B-NS5, appeared to reflect the large size of the gene segments. This suggests that a host range selection of mutations was probably not involved. By comparison, there was only one amino acid change in the entire genome of DEN-4 from MRC-5 cells whether or not serum was present in the medium. This finding indicates that a greater genomic stability of cloned DNA-derived DEN-4 was observed when the virus was propagated in MRC-5 cells, compared to virus propagated in Vero cells.

**Table 2:** Genomic sequences of the cloned DNA-derived DEN-4 viruses in Vero and MRC-5 cells on microcarriers, in serum-containing and serum-free media.

Vero cells					
DMEM+10%FBS			M-VSFM		
Gene	Mutation		Gene	Mutation	
	Nucleotide	Amino acid		Nucleotide	Amino acid
**C**	C228T	Silent			
**E**	A628G	K210E	**E**	T1443C	Silent
**NS1**	T923C	V308A	**NS1**	C366A	P122T
**NS1**	T946C	C316R	**NS1**	C777T	Silent
**NS2A**	T230C	M77T			
			**NS2B**	A156G	Silent
**NS3**	A27G	Silent	**NS3**	T240C	Silent
**NS3**	T793C	F265L	**NS3**	A625G	R209G
**NS3**	A1202G	D401G	**NS3**	A1320G	Silent
**NS3**	G1215A	Silent	**NS3**	A1348G	T450A
**NS3**	G1283A	C428Y			
**NS3**	A2202G	Silent			
**NS4A**	A330G	I110M	**NS4A**	T211G	F71V
			**NS4A**	T244C	S82P
**NS4B**	A336C	L112F			
**NS5**	A458T	N153I	**NS5**	A1708C	K569Q
**NS5**	G476A	G159E			
**NS5**	A816C	Silent			
**NS5**	A1186G	R396G			
**NS5**	G1517A	G506E			
**MRC-5 cells**					
**DMEM+10%FBS**			**M-VSFM**		
**Gene**	**Mutation**		**Gene**	**Mutation**	
	**Nucleotide**	**Amino acid**		**Nucleotide**	**Amino acid**
**NS5**	C406T	P136S	**E**	G1033A	E345K

### Sequences of multiple clones of three genomic fragments

Since the previous DEN-4 sequence determination was made from one DNA clone representing each of the four genome segments, to further investigate the genome stability of DEN-4 during passages in Vero cells and in MRC-5 cells, sequences of multiple clones were determined. The DNA-derived DEN-4 viruses were obtained in Vero cells or in MRC-5 cells at three consecutively passages after *in vitro* RNA transfection (as P1, P2, P3 samples). P1 and P2 viruses were harvested from cells grown in T flask cultures and the P3 passage virus was from a microcarrier culture. Multiple clone sequencings were conducted on the three genomic fragments (WE1/W02R, W28/W29R, W21/W23R) because most mutations identified earlier were localized in these fragments. These fragments are: (i) WE1/W02R, consisting of the full-length E gene and the NS1 gene portion encoding the first 152 amino acids; (ii) W28/W29R, consisting of part of NS3 gene encoding the amino acids 42–455; and (iii) W21/W23R, consisting of the entire NS4B gene and the NS5 gene portion encoding the first 180 amino acids. A total of 180 independent clones were obtained for sequence determination in this study.

No nucleotide mutation was found in the WE1/W02R (E-NS1) fragments of P1 and P2 passages from Vero or MRC-5 cells (Table 3). However, there were 7 nucleotide differences that resulted in 5 amino acid mutations in E protein at G_104_C, F_108_I, G_427_R, V_439_F, and V_463_L from Vero cells after three passages. Likewise, there were 3 nucleotide differences including 3 amino acid mutations in E protein at E_345_K, N_362_K, and G_427_R from MRC-5 cells (Table 3). The mutation frequencies of G_104_C and F_108_I in Vero cells and E_345_K in MRC-5 cells were 70%, 60%, and 50% respectively. In the W28/W29R (NS3) fragments, the P3 viruses from microcarrier cultures revealed 16 mutations resulting in 11 amino acid changes in Vero cells and 3 mutations resulting in 2 amino acid changes in MRC-5 cells. Each of these mutations in the NS3 protein occurred at a frequency of 10% except E_341_G at a frequency of 20% (Table 4). In the W21/W23R (NS4B-NS5) fragment, there were 11 mutations resulting in 10 amino acid changes in the P3 virus from Vero cells on microcarriers. The mutations were found at L_112_F and A_240_V in the NS4B protein at a frequency of 30%, while other mutations occurred at a frequency of 10%. Interestingly, there was only 1 nucleotide mutation resulting in 1 amino acid change in DEN-4 from MRC-5 cells grown on microcarriers at P3 (Table 5).

**Table 3:** Nucleotide and amino acid mutations in the WE1-W02R (E-NS1) genetic fragment of the cloned DNA-derived DEN-4 viruses propagated in Vero and MRC-5 cells at three consecutive passages using serum-free medium.

Passage	Cell	Virus gene segment	Mutation		
			Frequency*	Nucleotide position	Amino acid position
P1	Vero	-	0/10	No change	No change
P2	Vero	-	0/10	No change	No change
P3	Vero	E	7/10	G 310 T	G 104 C
P3	Vero	E	6/10	T 322 A	F 108 I
P3	Vero	E	2/10	G 1278 C	G 427 R
P3	Vero	E	1/10	G 1314 T	V 439 F
P3	Vero	E	1/10	G 1386 T	V 463 L
P3	Vero	E	1/10	G 1409 A	Silent
P3	Vero	E	4/10	T 1442 C	Silent
P1	MRC-5	-	0/10	No change	No change
P2	MRC-5	-	0/10	No change	No change
P3	MRC-5	E	5/10	G 1033 A	E 345 K
P3	MRC-5	E	1/10	C 1085 A	N 362 K
P3	MRC-5	E	1/10	G 1278 A	G 427 R

* 10 clones were sequenced in each case.

**Table 4 pone-0001810-t004:** Nucleotide and amino acid mutations in the W28-W29R (NS3) genetic fragment of the cloned DNA-derived DEN-4 viruses propagated in Vero and MRC-5 cells at three consecutive passages using serum-free medium.

Passage	Cell	Virus gene segment	Mutation		
			Frequency*	Nucleotide position	Amino acid position
P1	Vero	-	0/10	No change	No change
P2	Vero	-	0/10	No change	No change
P3	Vero	NS3	1/10	T 150 C	Silent
P3	Vero	NS3	1/10	G 161 A	R 54 K
P3	Vero	NS3	1/10	C 304 G	P 102 A
P3	Vero	NS3	1/10	G 376 T	V 126 L
P3	Vero	NS3	1/10	A 391 G	K 131 E
P3	Vero	NS3	1/10	A 625 G	R 209 G
P3	Vero	NS3	1/10	A 663 G	Silent
P3	Vero	NS3	1/10	A 799 G	T 267 A
P3	Vero	NS3	1/10	A 998 G	E 333 G
P3	Vero	NS3	1/10	A 1007 G	E 336 G
P3	Vero	NS3	1/10	T 1017 C	Silent
P3	Vero	NS3	1/10	G 1020 C	Silent
P3	Vero	NS3	2/10	A 1022 G	E 341 G
P3	Vero	NS3	1/10	T 1078 G	W 360 G
P3	Vero	NS3	1/10	A 1320 G	Silent
P3	Vero	NS3	1/10	A 1348 G	T 400 A
P1	MRC-5	-	0/10	No change	No change
P2	MRC-5	-	0/10	No change	No change
P3	MRC-5	NS3	1/10	C 633 G	Silent
P3	MRC-5	NS3	1/10	C 668 T	P 223 L
P3	MRC-5	NS3	1/10	G 909 T	R 303 S

* 10 clones were sequenced in each case.

**Table 5:** Nucleotide and amino acid mutations in the W21-W23R (NS4B-NS5) genetic fragment of the cloned DNA-derived DEN-4 viruses propagated in Vero and MRC-5 cells at three consecutive passages using serum-free medium.

Passage	Cell	Virus gene segment	Mutation		
			Frequency*	Nucleotide position	Amino acid position
P1	Vero	-	0/10	No change	No change
P2	Vero	-	0/10	No change	No change
P3	Vero	NS4B	1/10	C 192 T	Silent
P3	Vero	NS4B	1/10	G 253 A	D 85 N
P3	Vero	NS4B	3/10	A 336 C	L 112 F
P3	Vero	NS4B	3/10	C 719 T	A 240 V
P3	Vero	NS5	1/10	C 167 T	S 56 F
P3	Vero	NS5	1/10	C 238 T	L 80 F
P3	Vero	NS5	1/10	G 251 A	R 84 K
P3	Vero	NS5	1/10	A 270 C	M 90 L
P3	Vero	NS5	1/10	A 287 C	N 96 T
P3	Vero	NS5	1/10	A 296 G	E 99 G
P3	Vero	NS5	1/10	A 308 T	Y 103 F
P1	MRC-5	NS4B	1/10	C 202 T	P 101 L
P3	MRC-5	-	0/10	No change	No change

* 10 clones were sequenced in each case.

These results were then analyzed on the basis of amino acid mutations detected in each of the three fragments sequenced. The mean diversity and mean p-distance of amino acid mutations in each genomic fragment of 10 independent clones were calculated (Table 6). The numbers of amino acid mutations within the WE1/W02R, W28/W29R, W21/W23R fragments respectively were 17, 12, 13 from Vero cells, and 7, 2, 0 from MRC-5 cells. The values of mean diversity calculated for these three genomic fragments were 0.31 (WE1/W02R), 0.29 (W28/W29R), and 0.31 (W21/W23R) in Vero cells, as compared to 0.13 (WE1/W02R), 0.05 (W28/W29R), and 0 (W21/W23R) in MRC-5 cells. The values of mean diversity calculated from MRC-5 cells were at least 2 folds lowered than from Vero cells. Calculation of the p-distance based on pair wise comparisons of amino acid sequences between clones also showed the values of mean p-distance were 0.36 (range 0–0.70) in Vero cells compared to 0.19 (range 0–0.50) in MRC-5 cells in the WE1-W02R fragment; 0.56 (range 0–0.12) in Vero cells compared to 0.13 (range 0–0.50) in MRC-5 cells in the W28-W29R fragment; 0.50 (range 0–1.40) in Vero cells compared to 0 (range 0) in MRC-5 cells in the W21-W23R fragment. All of these data demonstrated that the cloned DEN4 propagated in MRC-5 cells were more stable than those propagated in Vero cells.

**Table 6:** The number of amino acid mutations and the mean diversity for the three genetic fragments of the cloned DNA-derived DEN-4 viruses propagated in Vero and MRC-5 cells in serum-free cultures.

Cell line	WE1-W02R (E-NS1)				W28-W29R (NS3)				W21-W23R (NS4B-NS5)			
	total no. of amino acids = 551				total no. of amino acids = 420				total no. of amino acids = 426			
	No. of mutations	Mean diversity (%)^a^	p-distance (%)^b^		No. of mutations	Mean diversity (%)^a^	p-distance (%)^b^		No. of mutations	Mean diversity (%)^a^	p-distance (%)^b^	
			Mean	Range			Mean	Range			Mean	Range
**Vero**	17	0.31	0.36	0–0.70	12	0.29	0.56	0–1.20	14	0.31	0.50	0–1.40
**MRC-5**	7	0.13	0.19	0–0.50	2	0.05	0.13	0–0.50	0	0.00	0.00	0–0.00

a The mean diversity is the number of substitutions divided by the total number of amino acids sequenced.

b p-distances were calculated by pair wise comparison of amino acid sequences between clones by the program MEGA.

## Discussion

This work has investigated the replication kinetics of four-serotype and cloned DNA-derived DEN viruses in Vero and MRC-5 cells. Vero (African green monkey kidney) cells are polyploidy (recently certified as cell hosts for producing DEN vaccines) and MRC-5 (human fetal lung fibroblast cells) are diploid. Both cell lines have been already used to develop several human vaccines [28], and Vero cells are currently used for candidate DEN vaccine production. However, viral yield and genetic stability of DEN viruses have not been systematically compared between Vero and MRC-5 cell lines.

High titers of DEN-1 (HAWAII) and DEN-2 (NGC) but relatively low titers of DEN-3 (H-87) and DEN-4 (H-241) were obtained in this study in either Vero cells or MRC-5 cells, although both cells were grown to a similar density on microcarrieres prior to virus infection. A possible explanation is that the DEN-4 (H-241) virus strain used was the non mouse-adapted strain rather than the prototype mouse-adapted strain since the non mouse-adapted strain was associated with two E protein mutations (Ile for Thr-155, Leu for Phe-401) [29]. This study obtained high titers (1∼2×10^6^ pfu/ml) of cloned DEN-4 (81466 strain) in both Vero cells and MRC-5 cells. The viral yield of cloned DEN-4 (81466 strain) produced in MRC-5 cells on microcarriers (at P3) was approximately 10 folds lower and had only one amino acid change compared to those in Vero cells with thirteen and six amino acid mutations in serum-free and serum-containing cultures, respectively (Table 2). Almost no P1 and P2 virus mutations in the Vero and MRC-5 cells grown in T flasks were found by sequencing of multiple clones (Tables 3∼[Table pone-0001810-t004] 5). The increased genetic mutations of cloned DEN4 observed in Vero cells were probably caused by passage number or high-density microcarrier culture rather than the increased virus yield.

Comparative mutation rate in this study was based only on the genomic sequences of the cloned DNA-derived DEN4 from passages 1 through 3, the latter representing microcarrier culture viruses. This study did not investigate the genomic characteristics of the non-cloned wild type viruses such as the DEN-1 (HAWAII), DEN-2 (NGC), DEN-3 (H-87), and DEN-4 (H-241) strains used for the virus yield studies in Figs S1, S2, S3 and S4. Thus, only the cloned DNA-derived DEN-4 virus started genetically more homogeneous was analyzed. The genomic changes in the cloned DEN-4 viruses in Table 2 indicate that the mutations occurred in C, E, NS1, NS2A, NS3, NS4A, NS4B, and NS5 but not in the 5′-NTR or 3′-NTR regions. This result is partly agreed with other related reports of mutations in C, M, E, NS2A, NS4B and NS5 regions for the DEN-2/DEN-4 chimeric virus [30], and in 5′ NTR, C, E, NS1, NS3, NS4A, NS4B and NS5 regions for the chimeric DEN-4/Japanese encephalitis virus [31]. Multiple cloning experiments showed that most mutations were at 10% frequency and occurred in the third passage (P3) using microcarrier cultures (Tables 3, 4, 5). The E-G_104_C, E-F_108_I in Vero cells and the E-E_345_K in MRC-5 cells had mutation rates exceeding 50% and the NS4B-L_112_F and NS4B-A_240_V in Vero cells had mutation rates of 30%. These results conflict with the high rate of NS3 gene mutation reported in a recent study of the full-genomic sequences of several DEN-3 clinical isolates [32]. Reportedly, NS4B is a key determinant of Vero cell adaptation for the intertypic-DEN chimeric viruses [21], [31]. Our experiments showed that the NS4B-L_112_F and NS4B-A_240_V mutations occur in Vero cells at a frequency of only 30% (Table 5), which partially correlates with other studies of infectious clone DEN viruses [22] and 5′-FU mutagenized DEN viruses [21]. However, the present report found that the E-G_104_C, E-F_108_I mutations in Vero cells and the E-E_345_K mutation in MRC-5 cells occurred at a higher frequency than the NS4B-L_112_F and NS4B-A_240_V mutations in Vero cells.

The DEN2-PDK-53-based chimeric viruses have recently been characterized by multiple plaque selection and spot/genomic sequencing [23]. The current data based on the first three passages have already demonstrated a better genetic stability in MRC-5 cells than in Vero cells. Prompted by reports of success in manufacturing the DEN2-PDK-53-based tetravalent vaccine in certified Vero cells [23], the authors are currently investigating the genetic stability of attenuated chimeric cloned viruses of four serotypes to high passage levels. Passage numbers are limited to less than 10 since live-attenuated vaccines require limited passage levels from the seed virus to prevent unsafe virus reversion. After all, the data obtained in this study at the third passage of serum-free microcarrier cultures have already shown that the DEN virus propagated in MRC-5 cells is genetically more stable than in Vero cells. Therefore, despite the acceptance of Vero cells for many candidate DEN vaccine productions, the current findings indicate that MRC-5 cells grown on microcarriers may offer a more stable system for DEN virus production. This study is apparently the first to demonstrate that MRC-5 cells are a potentially better cell host for live-attenuated DEN vaccine production. These findings may be helpful in developing a biomanufacturing process for DEN vaccine production using high-density microcarrier cultures.

## Materials and Methods

### Cells and Media

Vero cells, MRC-5 cells and BHK-21 cells were obtained from the Bioresource Collection and Research Center (BCRC), Food Industrial Research and Development Institute, Hsinchu, Taiwan. Vero cells and MRC-5 cells were grown in Dulbecco's Modified Essential Medium (DMEM) (Invitrogen) and BHK-21 cells were grown in Eagle's Minimum Essential Medium (EMEM) (Sigma) supplemented with 10% heat-inactivated fetal bovine serum (FBS) and 100 U/ml of penicillin G sodium-Streptomycin (Invitrogen). Vero cells and MRC-5 cells were also cultured in serum-free medium using M-VSFM serum free medium (Biogro) [24]–[27] plus 100 U/ml of penicillin G sodium-Streptomycin. No prior adaptation in reduced serum concentrations was required for serum free cultures. Trypsin inhibitor (GIBCO) was used at 0.25% for cell detachment to protect cell damage by trypsin treatment under serum-free conditions.

### Viruses

DEN-1 virus (Hawaii), DEN-2 virus (NGC), DEN-3 virus (H-87) and DEN-4 virus (H-241) were provided by the Center of Disease Control, Department of Health, Taiwan. Stock viruses were prepared from supernatants of infected C6/36 cells grown in Hank's MEM medium (GIBCO) plus supplements 6 days post-infection at 28°C [33]. The plasmid of DEN-4 strain 814669 contained the full-length genomic sequence [12]. The plasmid was first linearized by cleavage with restriction enzyme *Kpn* I and then added to a transcription reaction mixture (Promega kit) containing m^7^G(5′)ppp(5′)G (Merck) for cap addition at the RNA 5′-end. After incubation at 37°C for 1.5 hour, the RNA product was purified with TRIzol reagent (Invitrogen) according to manufacturer's instructions. Prior to RNA transfection, subconfluent Vero cells and MRC-5 cells in a 24-well plate were rinsed once with serum-free medium and then covered with 0.3 ml of DMEM medium per well. The transfection mixture was prepared by adding 4 µl of DMRIE-C reagent (Invitrogen) to 1 ml of DMEM, then mixing with 10 µg of the RNA product. The transfection mixture was added directly to cell monolayers. After 18 hours incubation at 37°C, either DMEM+10% FBS or M-VSFM medium were added to the well. Eight days after transfection, culture supernatants were collected. All virus stocks were stored at −80°C freezer for further analysis.

### Microcarrier cultures

Cytodex 1 microcarriers (Amersham Biosciences) were prepared according to manufacturer's instructions. Briefly, the Cytodex 1 microcarriers were immersed in phosphate buffer saline (PBS) for at least three hours and autoclaved for 15 min before each experiment. The autoclaved microcarriers were washed twice with the culture medium. Bellco spinner flasks (100-ml) were used in the experiments for a working volume of 50 ml. The spinner flasks were stirred at 60 rpm and placed inside an incubator with 5% CO_2_ at 37°C. Cells were detached from tissue culture flasks using trypsin-EDTA, and then transferred to a spinner flask containing 2g/l Cytodex 1 microcarriers. Starting cell densities were 3×10^5^ cells/ml in serum-containing cultures and 6×10^5^ cells/ml in serum-free cultures. The cell cultures were infected after 3 day inoculation and by replacing 70% of the medium with fresh medium containing the virus inoculum at a multiplicity of infection (MOI) of 0.01. Inoculation continued without further replacement with fresh medium or addition of supplements.

### Determination of cell density and virus titer

The number of cells attached to the microcarriers was determined by nuclei staining. Briefly, a 1-ml sample of the microcarrier culture was taken and centrifuged at 200 *g* for 5 min to remove the supernatant. The pellets were treated with 1 ml 0.1 M citric acid [containing 0.1% (w/v) crystal violet] and incubated at 37°C for 1 hour. The released nuclei were counted in a hemocytometer. The virus titer was measured by 10-fold serial dilutions of the culture supernatant in duplicate infections of BHK-21 cell monolayers in a 6-well plate. After 1 hour incubation at 37°C, 4 ml of medium containing 10% FBS and 1.1% methylcellulose (4 ml/well) was added to each well. Virus plaques were stained with naphthol blue black dye six days after incubation. The infectivity titer in plaque forming units (pfu) per ml was determined.

### Sequencing the entire genome of cloned DNA-derived DEN-4

Viral RNA was extracted from culture supernatants using TRIzol reagent (Invitrogen), and the cDNA synthesized by reverse transcription using Superscript II RTase (Invitrogen). The double-stranded DNA was then generated by polymerase chain reaction (PCR) using Platinum® *Pfx* DNA polymerase (Invitrogen). Four overlapping fragments that span the entire DEN genome were produced using 4 sets of forward and reverse primers: (i) W01/W02R, (ii) W03/W04R, (iii) W05/W06R, and (iii) W07/W08R as listed in Table S1. The DNA products were purified by Gel/PCR DNA fragments extraction kit (Geneaid, Taiwan), cloned into the pGEM T-easy vector (Promega). The nucleotide sequences of each of the four fragments cloned into the pGEM T-easy vector (from two upstream promoters SP6 and T7 at both ends of the vector) were determined by Mission Biotech Inc., Taipei, Taiwan. The cloned DNA segments that span the entire DEN genome were mapped using the software Lasergene version 6.00 (DNASTAR, Inc. USA) to determine the extent of overlapping sequences.

### Sequencing multiple clones of three DEN-4 fragments

Three DNA fragments were synthesized from DEN-4 RNA by RT-PCR using Platinum® *Pfx* DNA polymerase with their respective forward and reverse primer sets: (i) WE1/W02R, (ii) W28/W29R, and (iii) W21/W23R (see Table S1 for sequences and their genomic locations). Each of these DNA fragments was inserted into pGEM T-easy cloning vector to obtain 10 or more independent clones for sequence analysis. The sequences were aligned in the WE1/W02R region (1,653-bp), the W28/W29R region (1,260-bp), and the W21/W23R region (1,278-bp) using the program Lasergene version 6.00 to generate the consensus sequence for each of the three fragemnts. The mean diversity of amino acids was determined as the number of amino acid substitutions divided by the total number amino acids sequenced [32], [34], [35]. Pair wise comparisons of amino acid sequences between clones were performed to determine the mean and range of pair wise distance matrix (p-distance) using the program MEGA version 3.1 (Molecular Evolutionary Genetics Analysis, Pennsylvania State University, PA). The p-distance is the proportion of amino acid mutations at which the two sequences to be compared are different.

## Supporting Information

Table S1 Primers used in the genomic sequencing of cloned DNA-derived DEN-4 viruses in cell cultures.
(0.18 MB DOC)

Figure S1 Production of DEN-1 virus (HAWAII strain) from Vero and MRC-5 cells grown on 2g/l Cytodex 1 microcarriers: (A) Vero cell growth curves; (B) MRC-5 cell growth curves; (C) virus replication curves in Vero cells; (D) virus replication curves produced in MRC-5 cells.
(0.06 MB TIF)

Figure S2 Production of DEN-2 virus (NGC strain) from Vero and MRC-5 cells grown on 2g/l Cytodex 1 microcarriers. Symbols are the same as Fig.S1.
(0.06 MB TIF)

Figure S3 Production of DEN-3 virus (H-87 strain) from Vero and MRC-5 cells grown on 2g/l Cytodex 1 microcarriers. Symbols are the same as Fig. S1.
(0.06 MB TIF)

Figure S4 Production of DEN-4 virus (H-241 strain) from Vero and MRC-5 cells grown on 2g/l Cytodex 1 microcarriers. Symbols are the same as Fig. S1.
(0.06 MB TIF)

Figure S5 Production of cloned DNA-derived DEN4 from Vero and MRC-5 cells grown on 2g/l Cytodex 1 microcarriers. Symbols are the same as Fig. S1.
(0.06 MB TIF)
