# Supplementary material for: High Genetic Stability of Dengue Virus Propagated in MRC-5 Cells as Compared to the Virus Propagated in Vero Cells
Source: PLoS One. 2008 Mar 19;3(3):e1810. doi: 10.1371/journal.pone.0001810 (PMC2265545; doi:10.1371/journal.pone.0001810)
Supplement: Table S1 — Primers used in the genomic sequencing of cloned DNA-derived DEN-4 viruses in cell cultures. (0.18 MB DOC) [file pone.0001810.s001.doc]

**Table S1 Primers used in the genomic sequencing of cloned DNA-derived DEN-4 viruses in cell cultures.**

| Primer name | Primer sequence | Viral genome position* |
| --- | --- | --- |
| W01 | AGTTGTTAGTCTGTGTGGAC | 1-20 |
| W02-R | CAGCCTCGTGGTTGATCTAA | 2593-2612 |
| W03 | CGAGACTAGCGTCTGCAATA | 2542-2561 |
| W04-R | CCCTCTCGATGTCTTCTATTGG | 5514-5535 |
| W05 | AGCCATCTTCATGACCGCAA | 5453-5472 |
| W06-R | CCTGTCGAAGGAGGTTCATA | 8487-8506 |
| W07 | CAGCGATTGCAAGAAGAGCA | 8406-8425 |
| W08-R | AGAACCTGTTGGATCAACAAC | 10629-10649 |
| WE1 | ATTGGGCAAACAGGAATCCAG | 870-891 |
| W02-R | CAGCCTCGTGGTTGATCTAA | 2593-2612 |
| W21 | TAATAGCAGCCAACGAGATG | 6817-6836 |
| W23-R | TATGACTGTTGGCATGTAGG | 8116-8135 |
| W28 | AAACTCAGGTTGGAGTAGGG | 4621-4640 |
| W29-R | TGCTGGGTTCCTTCCTATTC | 5902-5921 |

*Nucleotide position of the DEN-4 infectious clone 2A. GenBank accession number is AF375822.

Table 1

Vero cells (PFU/ml)

|  | DEN-1  (HAWAII) | DEN-2  (NGC) | DEN-3  (H-87) | DEN-4  (H-241) | DEN-4  (DNA clone) |
| --- | --- | --- | --- | --- | --- |
| DMEM+10%FBS | (1.7 ± 0.1) x 10^6^ | (1.2 ± 0.0) x 10^7^ | (1.3 ± 0.1) x 10^4^ | (2.0 ± 0.2) x 10^3^ | (1.6 ± 0.3) x 10^7^ |
| M-VSFM | (4.0 ± 0.1) x 10^6^ | (2.2 ± 0.0) x 10^7^ | (3.0 ± 0.1) x 10^4^ | (2.9 ± 0.2) x 10^3^ | (1.4 ± 0.1) x 10^7^ |

MRC-5 cells (PFU/ml)

|  | DEN-1  (HAWAII) | DEN-2  (NGC) | DEN-3  (H-87) | DEN-4  (H-241) | DEN-4  (DNA clone) |
| --- | --- | --- | --- | --- | --- |
| DMEM+10%FBS | (6.0 ± 0.1) x 10^5^ | (6.8 ± 0.3) x 10^5^ | (4.5 ± 0.7) x 10^3^ | (2.5 ± 0.7) x 10^2^ | (1.1 ± 0.8) x 10^6^ |
| M-VSFM | (7.7 ± 0.1) x 10^5^ | (2.0 ± 0.1) x 10^6^ | (1.3 ± 0.3) x 10^4^ | (9.0 ± 2.8) x 10^2^ | (1.2 ± 0.1) x 10^6^ |

Table 2

Vero cells

MRC-5 cells

| **DMEM+10%FBS** | | |  | **M-VSFM** | | |
| --- | --- | --- | --- | --- | --- | --- |
| **Gene** | **Mutation** | |  | **Gene** | **Mutation** | |
|  | **Nucleotide** | **Amino acid** |  |  | **Nucleotide** | **Amino acid** |
| **C** | C228T | Silent |  |  |  |  |
| **E** | A628G | K210E |  | **E** | T1443C | Silent |
| **NS1** | T923C | V308A |  | **NS1** | C366A | P122T |
| **NS1** | T946C | C316R |  | **NS1** | C777T | Silent |
| **NS2A** | T230C | M77T |  |  |  |  |
|  |  |  |  | **NS2B** | A156G | Silent |
| **NS3** | A27G | Silent |  | **NS3** | T240C | Silent |
| **NS3** | T793C | F265L |  | **NS3** | A625G | R209G |
| **NS3** | A1202G | D401G |  | **NS3** | A1320G | Silent |
| **NS3** | G1215A | Silent |  | **NS3** | A1348G | T450A |
| **NS3** | G1283A | C428Y |  |  |  |  |
| **NS3** | A2202G | Silent |  |  |  |  |
| **NS4A** | A330G | I110M |  | **NS4A** | T211G | F71V |
|  |  |  |  | **NS4A** | T244C | S82P |
| **NS4B** | A336C | L112F |  |  |  |  |
| **NS5** | A458T | N153I |  | **NS5** | A1708C | K569Q |
| **NS5** | G476A | G159E |  |  |  |  |
| **NS5** | A816C | Silent |  |  |  |  |
| **NS5** | A1186G | R396G |  |  |  |  |
| **NS5** | G1517A | G506E |  |  |  |  |

| **DMEM+10%FBS** | | |  | **M-VSFM** | | |
| --- | --- | --- | --- | --- | --- | --- |
| **Gene** | **Mutation** | |  | **Gene** | **Mutation** | |
|  | **Nucleotide** | **Amino acid** |  |  | **Nucleotide** | **Amino acid** |
|  |  |  |  | **E** | G1033A | E345K |
| **NS5** | C406T | P136S |  |  |  |  |

Table 3

| Passage | Cell | Virus gene segment | Mutation | | |
| --- | --- | --- | --- | --- | --- |
|  |  |  | Frequency | Nucleotide position | Amino acid position |
| P1 | Vero | - | 0/10 | No change | No change |
| P2 | Vero | - | 0/10 | No change | No change |
| P3 | Vero | E | 7/10 | G 310 T | G 104 C |
| P3 | Vero | E | 6/10 | T 322 A | F 108 I |
| P3 | Vero | E | 2/10 | G 1278 C | G 427 R |
| P3 | Vero | E | 1/10 | G 1314 T | V 439 F |
| P3 | Vero | E | 1/10 | G 1386 T | V 463 L |
| P3 | Vero | E | 1/10 | G 1409 A | Silent |
| P3 | Vero | E | 4/10 | T 1442 C | Silent |
| P1 | MRC-5 | - | 0/10 | No change | No change |
| P2 | MRC-5 | - | 0/10 | No change | No change |
| P3 | MRC-5 | E | 5/10 | G 1033 A | E 345 K |
| P3 | MRC-5 | E | 1/10 | C 1085 A | N 362 K |
| P3 | MRC-5 | E | 1/10 | G 1278 A | G 427 R |

Table 4

| Passage | Cell | Virus gene segment | Mutation | | |
| --- | --- | --- | --- | --- | --- |
|  |  |  | Frequency | Nucleotide position | Amino acid position |
| P1 | Vero | - | 0/10 | No change | No change |
| P2 | Vero | - | 0/10 | No change | No change |
| P3 | Vero | NS3 | 1/10 | T 150 C | Silent |
| P3 | Vero | NS3 | 1/10 | G 161 A | R 54 K |
| P3 | Vero | NS3 | 1/10 | C 304 G | P 102 A |
| P3 | Vero | NS3 | 1/10 | G 376 T | V 126 L |
| P3 | Vero | NS3 | 1/10 | A 391 G | K 131 E |
| P3 | Vero | NS3 | 1/10 | A 625 G | R 209 G |
| P3 | Vero | NS3 | 1/10 | A 663 G | Silent |
| P3 | Vero | NS3 | 1/10 | A 799 G | T 267 A |
| P3 | Vero | NS3 | 1/10 | A 998 G | E 333 G |
| P3 | Vero | NS3 | 1/10 | A 1007 G | E 336 G |
| P3 | Vero | NS3 | 1/10 | T 1017 C | Silent |
| P3 | Vero | NS3 | 1/10 | G 1020 C | Silent |
| P3 | Vero | NS3 | 2/10 | A 1022 G | E 341 G |
| P3 | Vero | NS3 | 1/10 | T 1078 G | W 360 G |
| P3 | Vero | NS3 | 1/10 | A 1320 G | Silent |
| P3 | Vero | NS3 | 1/10 | A 1348 G | T 400 A |
| P1 | MRC-5 | - | 0/10 | No change | No change |
| P2 | MRC-5 | - | 0/10 | No change | No change |
| P3 | MRC-5 | NS3 | 1/10 | C 633 G | Silent |
| P3 | MRC-5 | NS3 | 1/10 | C 668 T | P 223 L |
| P3 | MRC-5 | NS3 | 1/10 | G 909 T | R 303 S |

Table 5

| Passage | Cell | Virus gene segment | Mutation | | |
| --- | --- | --- | --- | --- | --- |
|  |  |  | Frequency | Nucleotide position | Amino acid position |
| P1 | Vero | - | 0/10 | No change | No change |
| P2 | Vero | - | 0/10 | No change | No change |
| P3 | Vero | NS4B | 1/10 | C 192 T | Silent |
| P3 | Vero | NS4B | 1/10 | G 253 A | D 85 N |
| P3 | Vero | NS4B | 3/10 | A 336 C | L 112 F |
| P3 | Vero | NS4B | 3/10 | C 719 T | A 240 V |
| P3 | Vero | NS5 | 1/10 | C 167 T | S 56 F |
| P3 | Vero | NS5 | 1/10 | C 238 T | L 80 F |
| P3 | Vero | NS5 | 1/10 | G 251 A | R 84 K |
| P3 | Vero | NS5 | 1/10 | A 270 C | M 90 L |
| P3 | Vero | NS5 | 1/10 | A 287 C | N 96 T |
| P3 | Vero | NS5 | 1/10 | A 296 G | E 99 G |
| P3 | Vero | NS5 | 1/10 | A 308 T | Y 103 F |
| P1 | MRC-5 | NS4B | 1/10 | C 202 T | P 101 L |
| P3 | MRC-5 | - | 0/10 | No change | No change |

Table 6

| Cell line | WE1-W02R (E-NS1)  total no. of amino acids = 551 | | | | W28-W29R (NS3)  total no. of amino acids = 420 | | | | W21-W23R (NS4B-NS5)  total no. of amino acids = 426 | | | |
| --- | --- | --- | --- | --- | --- | --- | --- | --- | --- | --- | --- | --- |
|  | No. of mutations | Mean diversity (%)^a^ | p-distance (%)^b^ | | No. of mutations | Mean diversity (%)^a^ | p-distance (%)^b^ | | No. of mutations | Mean diversity (%)^a^ | p-distance (%)^b^ | |
|  |  |  | Mean | Range |  |  | Mean | Range |  |  | Mean | Range |
| Vero | 17 | 0.31 | 0.36 | 0-0.70 | 12 | 0.29 | 0.56 | 0-1.20 | 14 | 0.31 | 0.50 | 0-1.40 |
| MRC-5 | 7 | 0.13 | 0.19 | 0-0.50 | 2 | 0.05 | 0.13 | 0-0.50 | 0 | 0.00 | 0.00 | 0-0.00 |

^a^ The mean diversity is the number of substitutions divided by the total number of amino acids sequenced.

^b^ p-distances were calculated by pair wise comparison of amino acid sequences between clones by the program MEGA.
