# Supplementary figures and images for: High Genetic Stability of Dengue Virus Propagated in MRC-5 Cells as Compared to the Virus Propagated in Vero Cells
Source: PLoS One. 2008 Mar 19;3(3):e1810. doi: 10.1371/journal.pone.0001810 (PMC2265545; doi:10.1371/journal.pone.0001810)

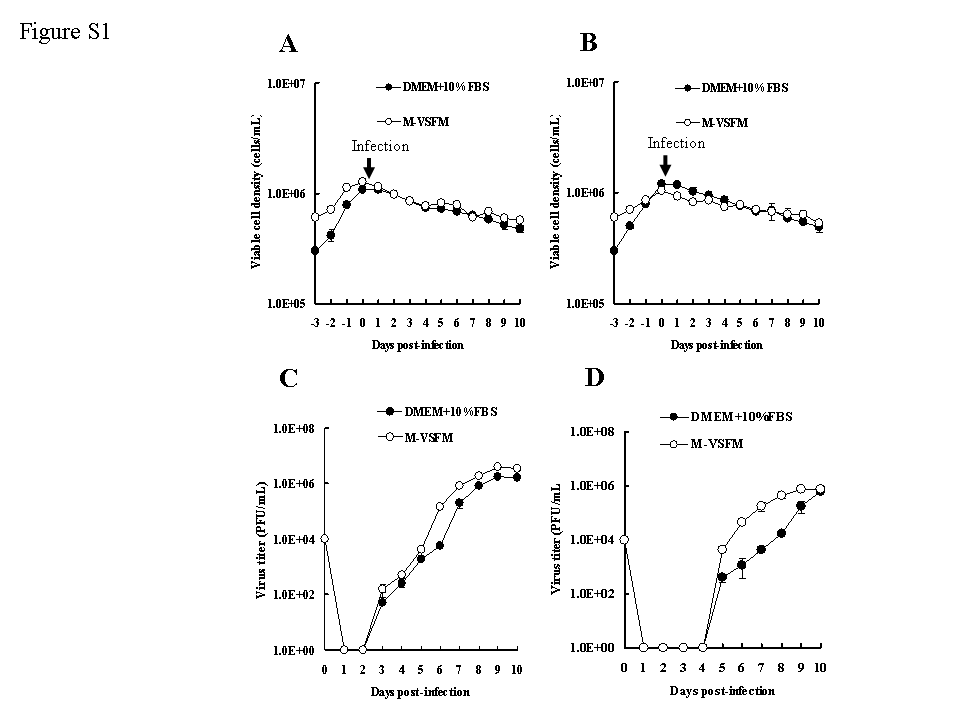

Supplement: Figure S1 — Production of DEN-1 virus (HAWAII strain) from Vero and MRC-5 cells grown on 2g/l Cytodex 1 microcarriers: (A) Vero cell growth curves; (B) MRC-5 cell growth curves; (C) virus replication curves in Vero cells; (D) virus replication curves produced in MRC-5 cells. (0.06 MB TIF) [file pone.0001810.s002.tif]

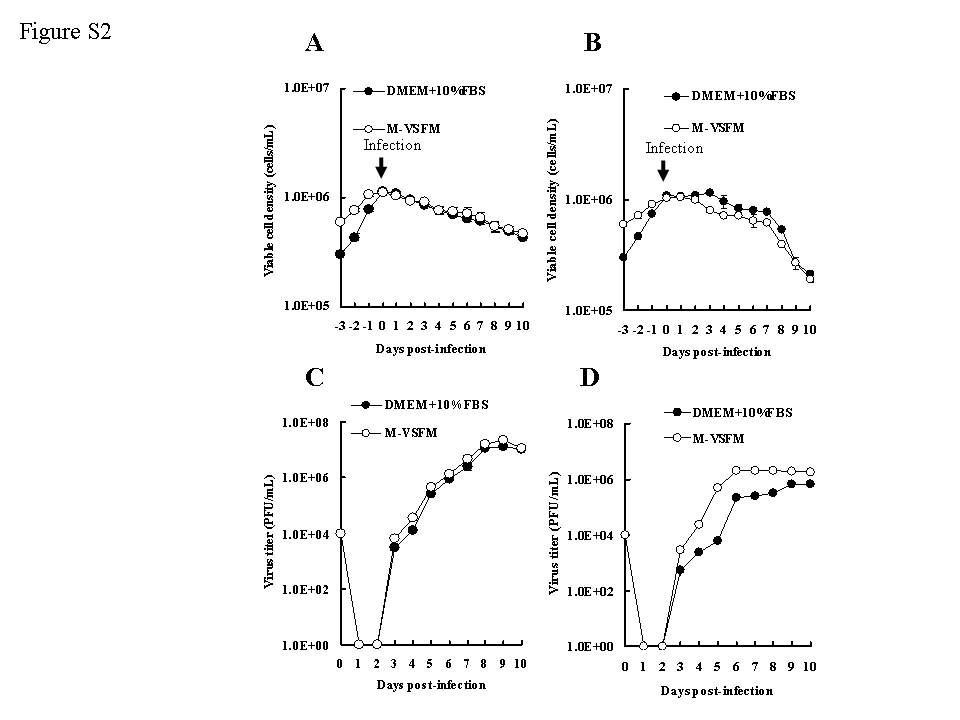

Supplement: Figure S2 — Production of DEN-2 virus (NGC strain) from Vero and MRC-5 cells grown on 2g/l Cytodex 1 microcarriers. Symbols are the same as Fig.S1. (0.06 MB TIF) [file pone.0001810.s003.tif]

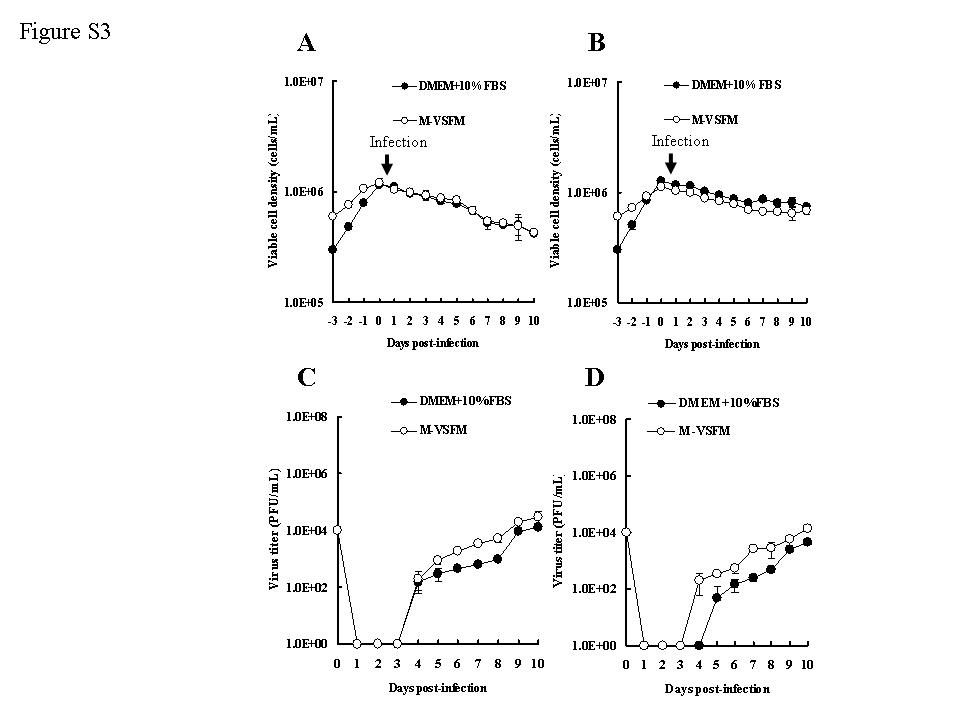

Supplement: Figure S3 — Production of DEN-3 virus (H-87 strain) from Vero and MRC-5 cells grown on 2g/l Cytodex 1 microcarriers. Symbols are the same as Fig. S1. (0.06 MB TIF) [file pone.0001810.s004.tif]

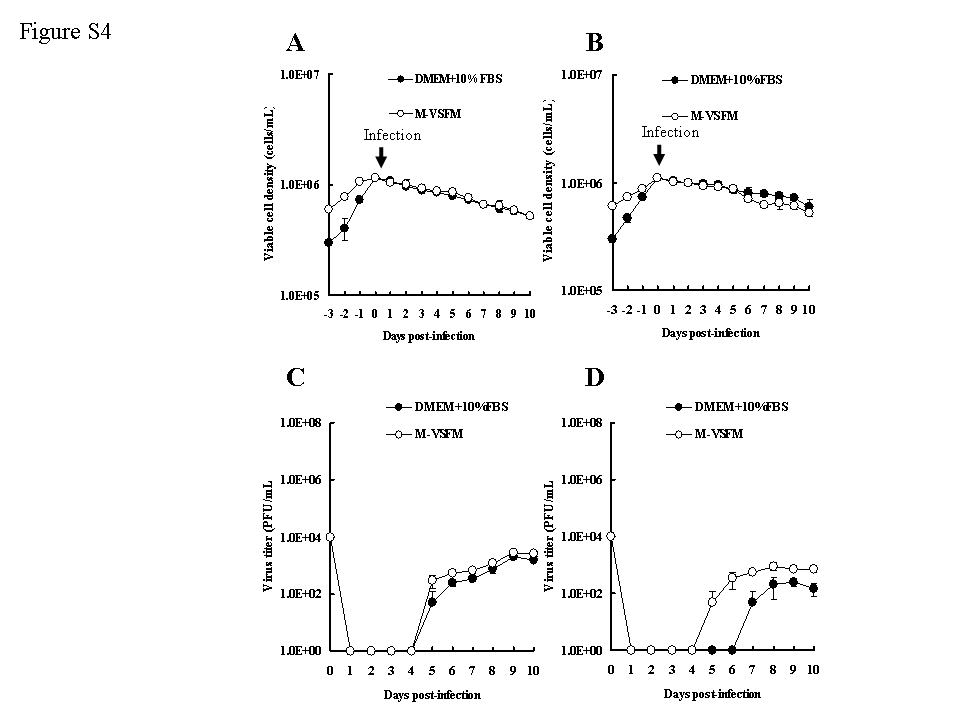

Supplement: Figure S4 — Production of DEN-4 virus (H-241 strain) from Vero and MRC-5 cells grown on 2g/l Cytodex 1 microcarriers. Symbols are the same as Fig. S1. (0.06 MB TIF) [file pone.0001810.s005.tif]

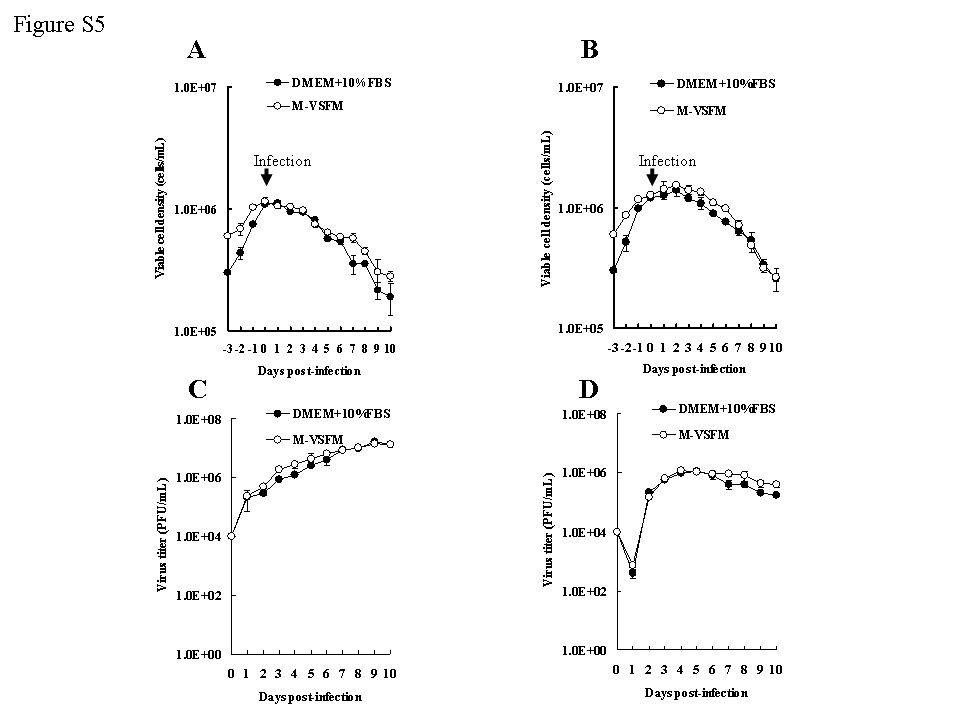

Supplement: Figure S5 — Production of cloned DNA-derived DEN4 from Vero and MRC-5 cells grown on 2g/l Cytodex 1 microcarriers. Symbols are the same as Fig. S1. (0.06 MB TIF) [file pone.0001810.s006.tif]
